# Supplementary material for: Algal exudates promote conjugation in marine Roseobacters
Source: mBio. 2024 Aug 27;15(10):e01062-24. doi: 10.1128/mbio.01062-24 (PMC11481893; doi:10.1128/mbio.01062-24)
Supplement: Supplemental Figures and Tables — Figures S1-S8; Tables S1-S8. [file mbio.01062-24-s0001.pdf]

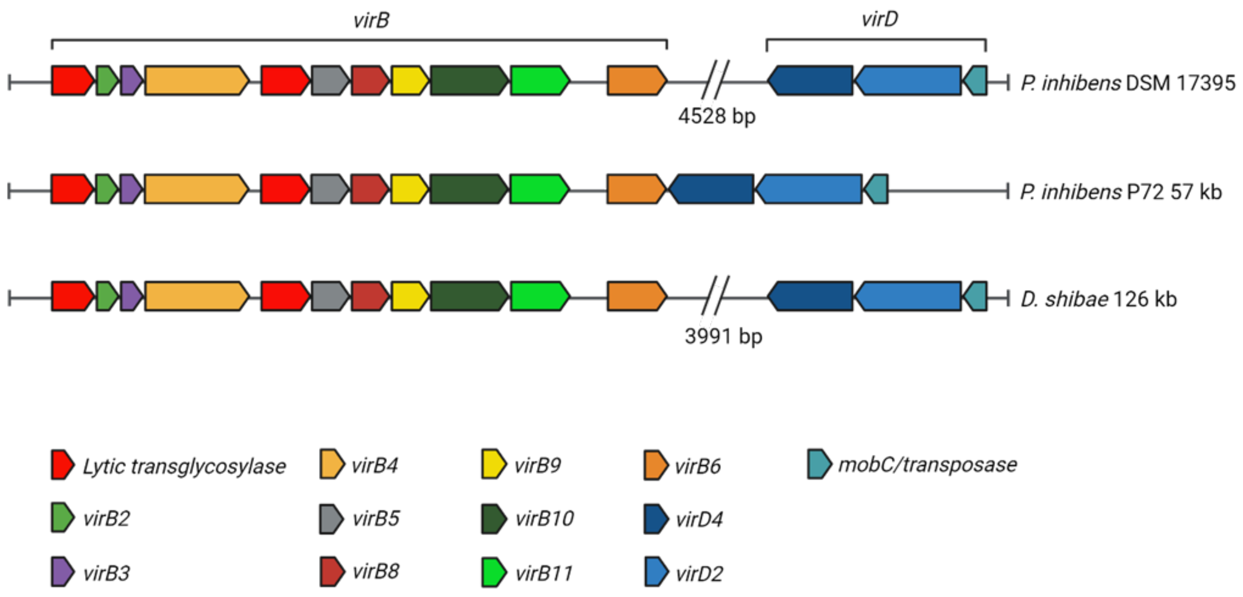

Fig. S1. Genetic organization of the T4SS genes in the donor strains. Schematic representation of genes from the *virB* and *virD* operons, which construct the T4SS, located in the chromosome of *P. inhibens* DSM 17395, the 57 kb plasmid of *P. inhibens* P72 and the 126 kb plasmid *of D. shibae*. Homologous genes are shown in the same color code. Of note, a potentially functional conjugative T4SS (*i.e.,* complete T4SS) was determined as one that includes all the *vir* genes that comprise the T4SSs located on the conjugative plasmids of *D. shibae* and *P. inhibens* P72.

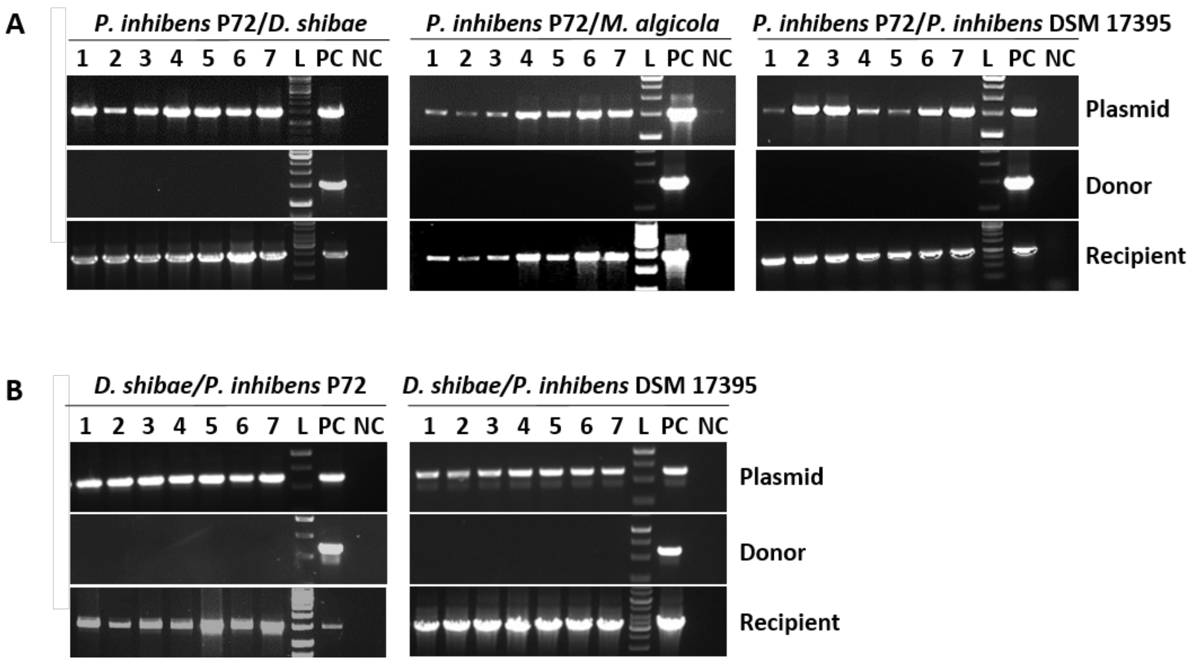

Fig. S2. Validation of double-resistance colonies from conjugation assays as transconjugants. Gel images of PCR products for validation of the transconjugants. **A)** *P. inhibens* P72 $^{p57\text{-}kana}$ as a donor strain, *D. shibae* DFL-12 $^{chr\text{-}gent}$, *M. algicola* DG898 $^{chr\text{-}gent}$, and *P. inhibens* DSM 17395$^{chr\text{-}gent}$ as recipients**. B)** *D. shibae* DFL-12 $^{p126\text{-}gent}$ as a donor strain, *P. inhibens* DSM 17395$^{chr\text{-}kana}$ as recipient. From each donor-recipient pair (Fig. 2), seven transconjugants colonies were randomly selected (marked 1-7). Each colony was streaked three times, followed by genomic extraction and PCR reactions. The transconjugants were tested using three sets of primers that identify the donor strain, recipient strain, and the transferred plasmid. Positive controls (PC) included primers and the corresponding strain DNA, and negative controls (NC) included the opposing strain DNA (i.e., primers targeting the donor strain with DNA of the recipient strain). The title above each graph denotes *Donor/Recipient*. L marks the 1 kb ladder incorporated in all gels

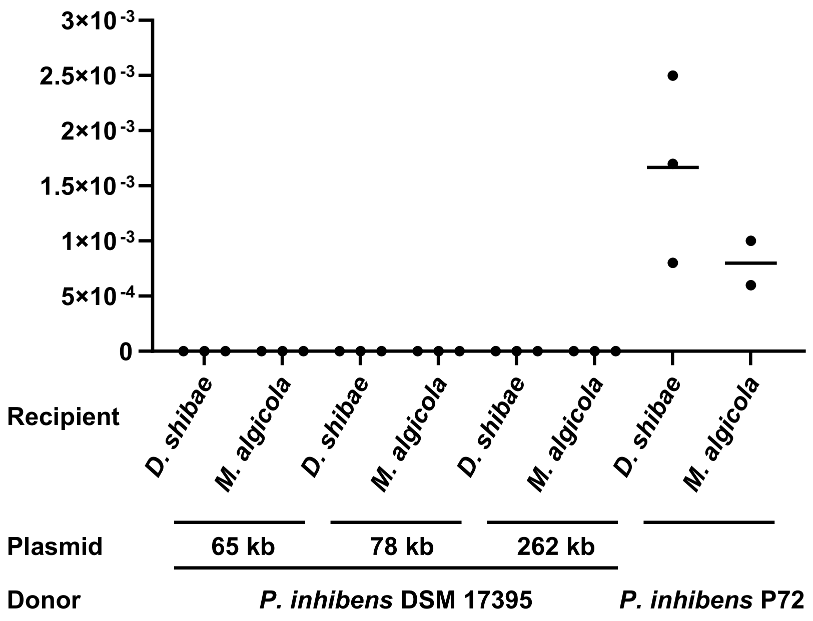

Fig. S3. The *P. inhibens* strain DSM 17395 is unable to serve as a plasmid donor. Results of conjugations assays performed on MB agar plates. Conjugation assays were carried out using *P. inhibens* DSM 17395 as the donor, with a kanamycin-resistance gene on one of its native 65 kb, 78 kb and 262 kb plasmids (*P. inhibens* DSM 17395 $^{p65\text{-}gent}$, *P. inhibens* DSM 17395 $^{p78\text{-}gent}$, and *P. inhibens* DSM 17395 $^{p262\text{-}gent}$ respectively). Each donor strain was mated with recipient strains *M. algicola* DG898 $^{chr\text{-}gent}$ or *D. shibae* DFL-12 $^{chr\text{-}gent}$, marked with a gentamicin-resistance gene on their chromosome. As a control, *P. inhibens* P72 $^{p57\text{-}kana}$, carrying a kanamycin-resistance gene on its 57 kb native plasmid was used as a donor and mated with the same recipients. Dots represent individual biological replicate and lines indicate the mean value. Conjugation efficiency was calculated by normalizing transconjugant cell numbers to donor cell numbers.

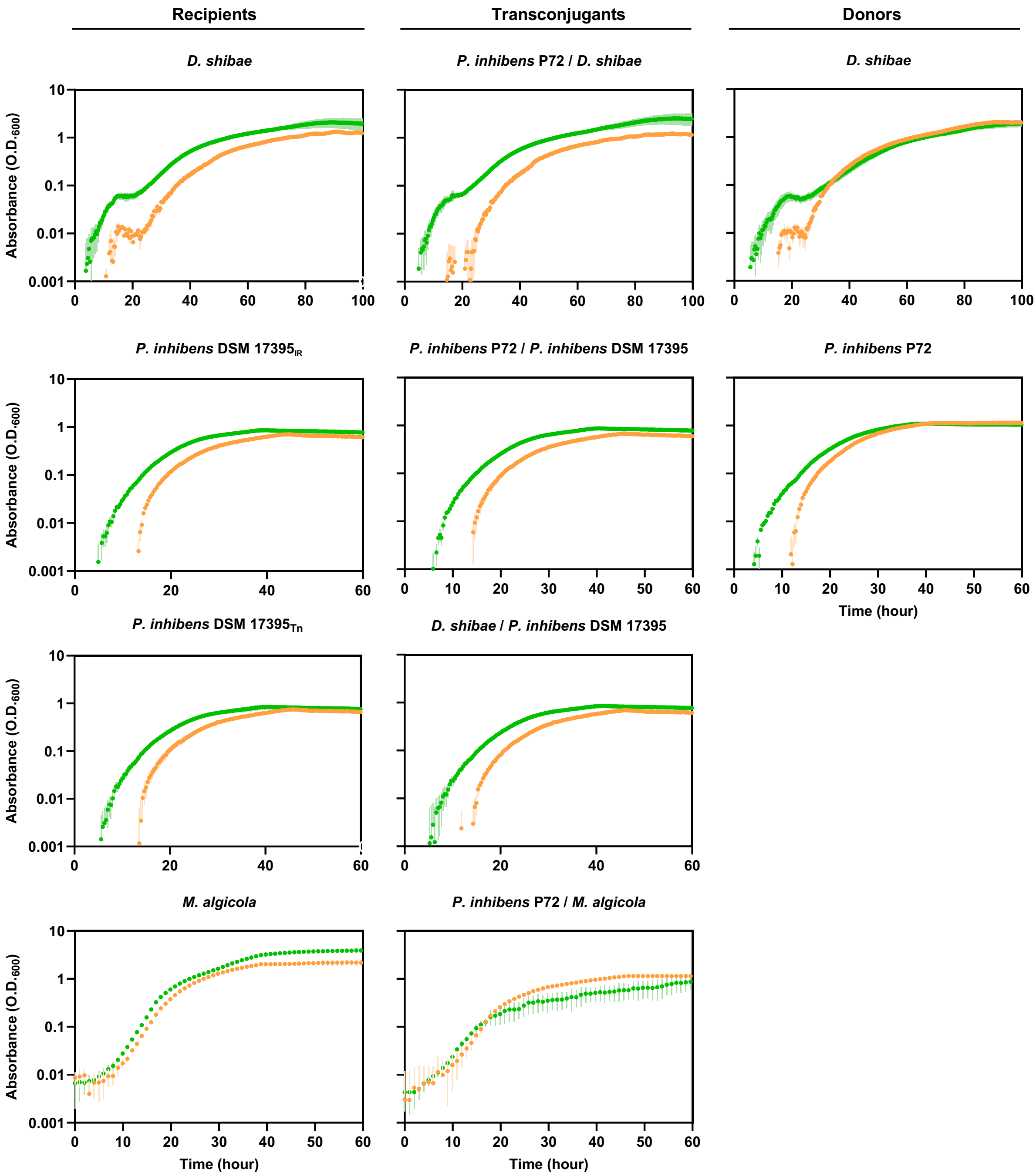

Fig. S4. Algal filtrates effect on bacterial growth. Bacterial cultures of recipients (left column), transconjugants (middle column) and donors (right column), were treated with either CNS medium as control conditions (orange dots) or with algal filtrates (green dots). Growth was evaluated by measuring the optical density using a wavelength of 600 nm (O.D.$_{600}$). Each dot represents the average of three biological replicates, error bars designate ± SD. The title above the column of transconjugants denotes *Donor/Recipient.*

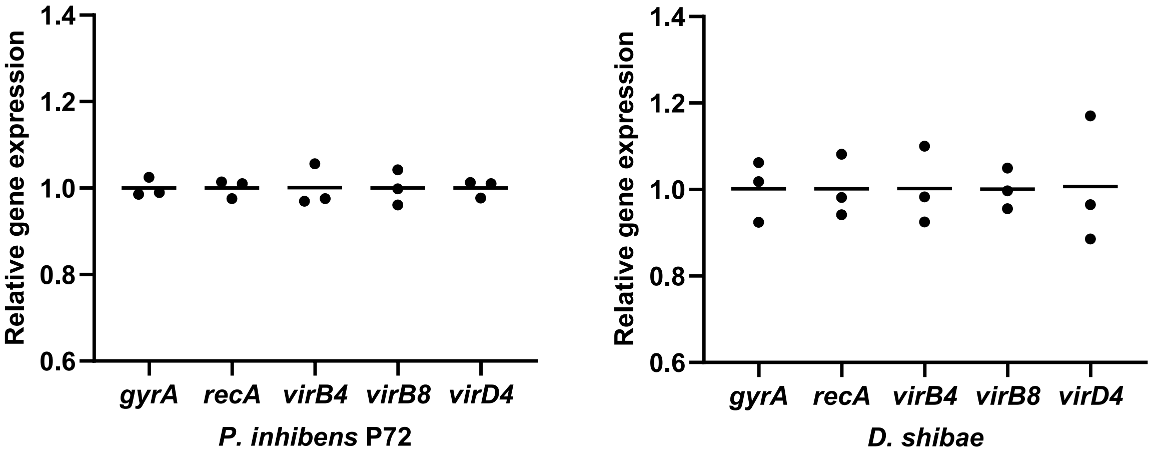

Fig. S5. Expression of T4SS genes is comparable to those of the housekeeping genes. Relative gene expression of *virB4*, *virB8* and *virD4* genes was assessed using qRT-PCR and compared to the house keeping genes *gyrA* and *recA*. RNA was extracted from bacterial cultures of *D. shibae* DFL-12 $^{chr\text{-}gent}$ and *P. inhibens* P72 $^{p57\text{-}kana}$ grown in CNS medium, when they reached OD_{600} of 0.3. Data points represent the mean of three technical replicates originating from one biological sample, lines represent replicates mean values.

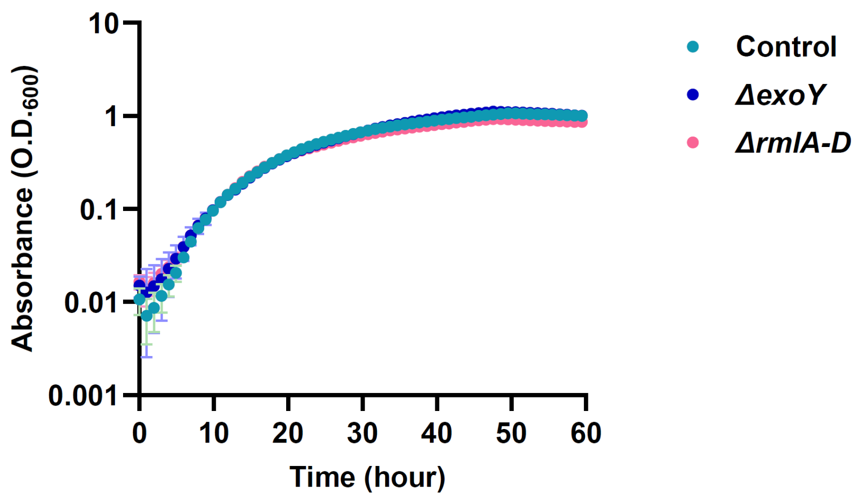

Fig S6. Growth curves of mutants with reduced attachment capabilities. Growth of *P. inhibens* DSM 17395 strains. Control (WT, blue), *ΔexoY* (green) and *ΔrmlA-D* (pink) were grown in CNS medium. Growth was assessed by measuring the optical density at a wavelength of 600 nm (O.D.$_{600}$). Each dot represents the average of three biological replicates, error bars designate ± SD.

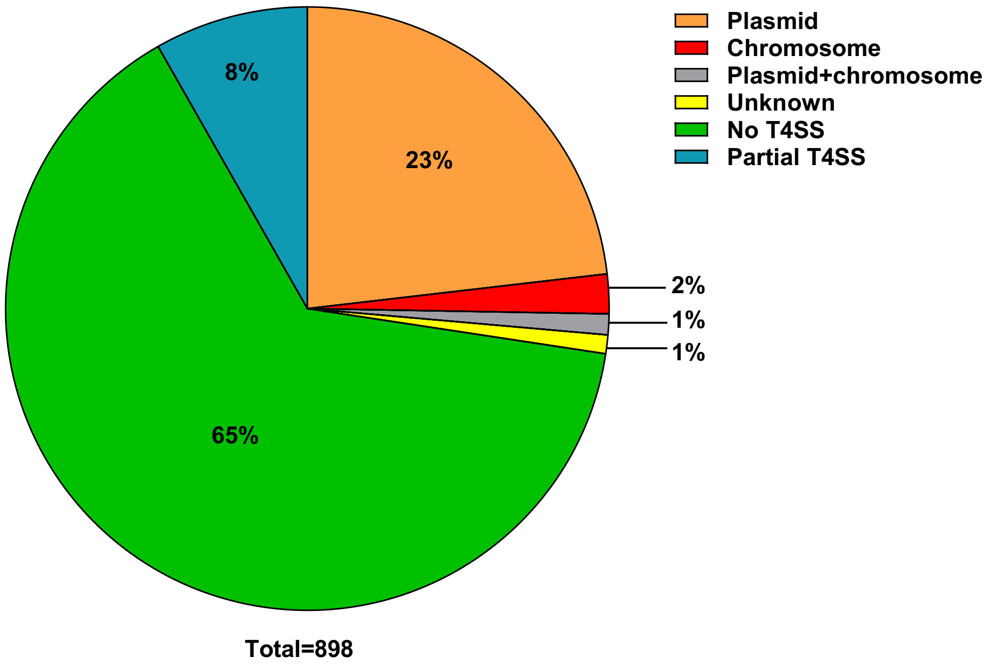

Fig. S7. Chromosome-encoded and plasmids-encoded T4SSs in Roseobacter bacteria. Roseobacter bacteria genomes were searched for T4SSs, and percentages were calculated for genomes with no T4SSs (green), partial T4SSs (blue), complete plasmid-encoded T4SSss (orange), complete chromosome-encoded T4SSs (red), complete plasmid-encoded and chromosome-encoded T4SSs located in the same genome (grey), and complete T4SSs with an unknown locus (yellow). Of note, a potentially functional conjugative T4SS (*i.e.,* complete T4SS) was determined as one that includes all the *vir* genes that comprise the T4SSs located on the conjugative plasmids of *D. shibae* and *P. inhibens* P72.

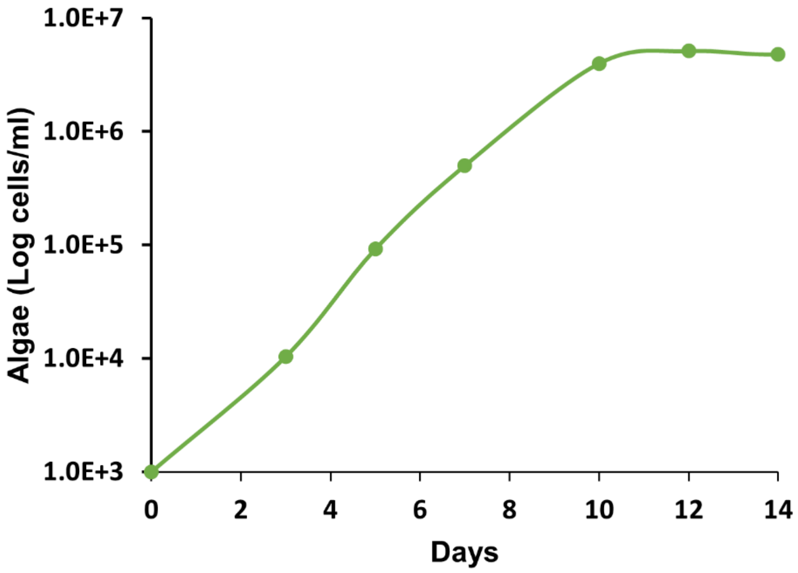

Fig. S8. Growth curves of axenic *Emiliania huxleyi* cultures. The cultures were cultivated at 18°C in ASW with L1 supplements, and monitored for 14 days, with spent media collected on day 12. Each data point in the growth curve represents the average from three biological replicates. Error bars indicate the standard deviation.

Table S1: List of bacterial strains and mutants.

Strain	Tagged locus	Insertion site	Reference	Lab collection identifier	Resistance	Comments
Phaeobacter inhibens DSM 17395			DSMZ, Germany	ES1		Wild type
Phaeobacter inhibens DSM 17395	65 kb plasmid	18,622	Current study	ES172	Kanamycin	Intergenic region mutant.
Phaeobacter inhibens DSM 17395	78 kb plasmid	26,249-30,284	Lab collection	ES171	Kanamycin	Knock-out mutant in both PGA1_RS19420 and PGA1_RS194 15.
Phaeobacter inhibens DSM 17395	262 kb plasmid	138,002	Dr. Jörn Petersen from the Leibniz Institute, DSMZ, Germany.	ES170	Kanamycin	Insertion in PGA1_262p01260, generated with the <R6kyori/KAN-2> transposon-kit.
Dinoroseobac ter shibae DFL-12			DSMZ, Germany	ES9		Wild type
Dinoroseobac ter shibae DFL-12	126 kb plasmid	27,457	Patzelt *et al.*[23]	ES94	Gentamicin	Insertion in Dshi_3964, generated using mariner transposon mutagenesis.
Dinoroseobac ter shibae DFL-12	Chromosome	3,502,458	Birmes *et al.*[22]	ES95	Gentamicin	Intergenic region mutant, generated using mariner transposon mutagenesis.
Phaeobacter inhibens P72			Dr. Jörn Petersen from the Leibniz Institute, DSMZ, Germany and Prof. Juan Barja from University of Santiago de Compostel a, Spain	ES98		Wild type
Phaeobacter inhibens P72	57 kb plasmid	38,628	Birmes *et al.*[22]	ES96	Kanamycin	Insertion in CP010740, generated with the <R6kyori/KAN-2> transposon-kit.
Phaeobacter inhibens P72	Chromosome	1,678,662	Dr. Jörn Petersen from the Leibniz Institute, DSMZ, Germany	ES168	Kanamycin	Insertion in PhaeoP72_RS079 95, generated with the <R6kyori/KAN-2> transposon-kit.
Marinovum algicola DG898	Chromosome	3,256,562	Birmes *et al.*[22]	ES93	Gentamicin	Insertion in MALG_03188, generated using

						mariner transposon mutagenesis.
Phaeobacter inhibens DSM 17395	Chromosome	1,400,112	Lab collection	ES70	Gentamicin	Intergenic region mutant.
Phaeobacter inhibens DSM 17395	Chromosome	2,372,826	Dr. Jörn Petersen from the Leibniz Institute, DSMZ, Germany.	ES97	Kanamycin	Insertion in PGA1_RS11385, generated with the <R6kyori/KAN-2> transposon-kit.
Escherichia coli ST18 DSM 22074			DSMZ, Germany	ES201		
Phaeobacter inhibens DSM 17395	65 kb plasmid	28,487-32,445	Current study	ES203	Kanamycin	Rhamnose operon knock-out mutant in genes: PGA1_65p00220, PGA1_65p00230, PGA1_65p00240 and PGA1_65p00250
Phaeobacter inhibens DSM 17395	Chromosome	521,726	Current study	ES208	Kanamycin	*exoY* knock-out mutant in PGA1_RS02605
Phaeobacter inhibens DSM 17395	Chromosome	2,858,218-2,859,345	Lab collection	ES138	Kanamycin	*metE* knock-out mutant in PGA1_c27430

Table S2: List of donor-recipient conjugation pairs. Donors are denoted by their transferred plasmid size in kilobases (Kb) represented as “p” for plasmid, and the antibiotic resistance gene that marks the plasmid (“gent” for gentamicin-resistance and “kana” for kanamycin-resistance). Recipients are designated as “chr” and the antibiotic resistance gene that marks their chromosome.

Donor	Recipient
P. inhibens DSM 17395 $^{\text{p262-kana}}$	*D. shibae* DFL-12 $^{\text{chr-gent}}$
P. inhibens DSM 17395 $^{\text{p78-kana}}$	*D. shibae* DFL-12 $^{\text{chr-gent}}$
P. inhibens DSM 17395 $^{\text{p65-kana}}$	*D. shibae* DFL-12 $^{\text{chr-gent}}$
P. inhibens DSM 17395$^{\text{p262-kana}}$	*M. algicola* DG898 $^{\text{chr-gent}}$
P. inhibens DSM 17395 $^{\text{p78-kana}}$	*M. algicola* DG898 $^{\text{chr-gent}}$
P. inhibens DSM 17395 $^{\text{p65-kana}}$	*M. algicola* DG898 $^{\text{chr-gent}}$
P. inhibens P72 $^{\text{p57-kana}}$	*D. shibae* DFL-12 $^{\text{chr-gent}}$
P. inhibens P72 $^{\text{p57-kana}}$	*M. algicola* DG898 $^{\text{chr-gent}}$
P. inhibens P72 $^{\text{p57-kana}}$	*P. inhibens* DSM 17395 $^{\text{chr-gent}}$
D. shibae DFL-12 $^{\text{p126-gent}}$	*P. inhibens* DSM 17395 $^{\text{chr-kana (ES97)}}$
D. shibae DFL-12 $^{\text{p126-gent}}$	*P. inhibens* P72 $^{\text{chr-kana}}$
D. shibae DFL-12 $^{\text{p126-gent}}$	*P. inhibens* DSM 17395 $^{\text{chr-kana (ES138)}}$
D. shibae DFL-12 $^{\text{p126-gent}}$	*P. inhibens* DSM 17395 $^{\Delta \textit{exoY}\text{-kana}}$
D. shibae DFL-12 $^{\text{p126-gent}}$	*P. inhibens* DSM 17395 $^{\Delta \textit{rmlA-D}\text{-kana}}$

Table S3: Primers for transconjugant validation. Each transconjugant was validated using three primer sets identifying the donor, the recipient, and the transferred plasmid. Primer sequences are listed in table 5. Donors are denoted by their transferred plasmid size in kilobases (Kb) represented as “p” for plasmid, and the antibiotic resistance gene that marks the plasmid (“gent” for gentamicin-resistance and “kana” for kanamycin-resistance). Recipients are designated as “chr” and the antibiotic resistance gene that marks their chromosome.

Strain	Target of the primer set	Primer name
D. shibae DFL-12 $^{p126\text{-}gent}$	Donor	802+803
P. inhibens P72 $^{p57\text{-}kana}$	Donor	794+777
P. inhibens DSM 17395$^{chr\text{-}kana}$	Recipient	126+92
D. shibae DFL-12 $^{chr\text{-}gent}$	Recipient	802+803
M. algicola DG898 $^{chr\text{-}gent}$	Recipient	804+805
P. inhibens DSM 17395$^{chr\text{-}gent}$	Recipient	47+290
P. inhibens P72 $^{chr\text{-}kana}$	Recipient	796+797
D. shibae DFL-12, 126 kb plasmid	plasmid	772+773
P. inhibens P72, 57 kb plasmid	plasmid	808+809

Table S4: Primer list for PCR reactions and construction of mutant strain. Chromosomally marked strains are designated as "chr". Antibiotic resistance markers are indicated by "gent" for gentamicin-resistance and "kana" for kanamycin-resistance.

Primer name	Sequence	Strain	Comments	Origin
47	CAGGATGAGTTATTGCACCA	*P. inhibens* DSM 17395$^{chr\text{-}gent}$	Validation of transconjugants	Current study
290	GAGCCTACATGTGCGAATGATGC	*P. inhibens* DSM 17395$^{chr\text{-}gent}$	Validation of transconjugants	Current study
92	CCAGAGAACAATAGTAAGAGCG	*P. inhibens* DSM 17395$^{chr\text{-}kana}$	Validation of transconjugants	Current study
126	GCTTGATGTCGAGGTGCTGT	*P. inhibens* DSM 17395$^{chr\text{-}kana}$	Validation of transconjugants	Current study
772	GGCACCATCGTCGGAACCAAT	*D. shibae* DFL-12, 126 kb plasmid	Validation of transconjugants	Patzelt *et al.*[23]
773	TGGTATCAGGCATTCGCTTCA	*D. shibae* DFL-12, 126 kb plasmid	Validation of transconjugants	Patzelt *et al.*[23]
777	AGATCGCCAAATTGCAGCAG	*P. inhibens* P72	Validation of transconjugants	Current study
794	AGACGACCCCTTGAGGCAC	*P. inhibens* P72	Validation of transconjugants	Current study
802	ACGGCATCGACCACTTCTTC	*D. shibae* DFL-12	Validation of transconjugants	Birmes *et al.*[22]
803	ACGGAAGATCGGGTACATGG	*D. shibae* DFL-12	Validation of transconjugants	Birmes *et al.*[22]
804	CAATCCGATCGACAGCTACG	*M. algicola* DG898	Validation of transconjugants	Birmes *et al.*[22]
805	TGATCGGCATGTAGAGCAGC	*M. algicola* DG898	Validation of transconjugants	Birmes *et al.*[22]
808	GAACCAGGAATGCATCGGTC	*P. inhibens* P72, 57b kb plasmid	Validation of transconjugants	Birmes *et al.*[22]
809	CCTGATCCGGCTATGACCAT	*P. inhibens* P72, 57b kb plasmid	Validation of transconjugants	Birmes *et al.*[22]
974	GCGAATTTTAACAAAATATTAACG CTTACATATGTGCAGCCTTCTCAA CCTGAGG	*D. shibae* DFL-12	191 kb plasmid reporter strain	Current study
975	CACCAGTGAACAGCTCTTCGCCT TTACGCATCTGCGCATTTGCGGA ACCCGTCAGGAACCAG	*D. shibae* DFL-12	191 kb plasmid reporter strain	Current study
972	CGCGAATTTTAACAAAATATTAAC GCTTACACCAAACTTGCGCAGCG AGGAAGCGTCTC	*D. shibae* DFL-12	126 kb plasmid reporter strain	Current study
973	CACCAGTGAACAGCTCTTCGCCT TTACGCATTTGCGCAGTGGCGGG ACCCGCGAGATACCAG	*D. shibae* DFL-12	126 kb plasmid reporter strain	Current study
987	GCGAATTTTAACAAAATATTAACG CTTACATGCCACCCATCGGCTCA ACCTTAAGTTTCTG	*P. inhibens* P72	184 kb plasmid reporter strain	Current study
988	CACCAGTGAACAGCTCTTCGCCT TTACGCATCTGCGCATTGGCGGA ACCCGTCAGGAACCAG	*P. inhibens* P72	184 kb plasmid reporter strain	Current study
989	GCGAATTTTAACAAAATATTAACG CTTACAGTAGCCCGCTCTAGCCC GTTGGGTCATCCTG	*P. inhibens* P72	57 kb plasmid reporter strain	Current study
990	CACCAGTGAACAGCTCTTCGCCT TTACGCATGCCCTGAGCCAGAGC TACGGTGCAGGAGAGC	*P. inhibens* P72	57 kb plasmid reporter strain	Current study
132	CGTAGCACCAGGCGTTTAAGG	pBBR1MCS-5 plasmid	Validation of reporter strains	Current study
255	ACTTGAAGAAGTCATGCTGC	sfGFP protein	Validation of reporter strains	Current study

1169	GTACAAAAAAGCAGGCTCCGAATTCGCCCTTAACCAGTCCTTCCGCAACAAGACGCGCAAGG	*P. inhibens* DSM 17395	Construction of the 65 kb plasmid mutant	Current study
1170	CAGTTTACGAACCGAACAGGCTTATGTCAAGCGAAATATCACTATCTCCACACTACCACC	*P. inhibens* DSM 17395	Construction of the 65 kb plasmid mutant	Current study
1171	CTTCTATCGCCTTCTTGACGAGTTCTTCTGAGAGGATTAATTTAAATCGCACTCTTATTCTC	*P. inhibens* DSM 17395	Construction of the 65 kb plasmid mutant	Current study
1172	CTTTGTACAAGAAAGCTGGGTCGAATTCGCCCTACCATTCATAAGCACGTGCTGTGGAAATGTC	*P. inhibens* DSM 17395	Construction of the 65 kb plasmid mutant	Current study
1274	TGTACAAGAAAGCTGGGTCGAATTCGCCCTAGATCAATCCGGCTGTTGGTCTCAATATCG	*P. inhibens* DSM 17395	Construction of the Δ*exoY* mutant	Current study
1275	CAGTTTACGAACCGAACAGGCTTATGTCAATTTGCCTGCCGCTCTGCCCTGCGTGGGTC	*P. inhibens* DSM 17395	Construction of the Δ*exoY* mutant	Current study
1276	CTTCTATCGCCTTCTTGACGAGTTCTTCTGATCAGGCTGAGCGCACAATGCGCGTCAGGC	*P. inhibens* DSM 17395	Construction of the Δ*exoY* mutant	Current study
1277	TACAAAAAAGCAGGCTCCGAATTCGCCCTTATGGGTTTGTGGTTGATTTCTGACCGTGAAGC	*P. inhibens* DSM 17395	Construction of the Δ*exoY* mutant	Current study
1279	GTACAAGAAAGCTGGGTCGAATTCGCCCTATCACGGCAGTGACATCCGCCATCAACTCG	*P. inhibens* DSM 17395	Construction of the Δ*rmlA-D* mutant	Current study
1280	CAGTTTACGAACCGAACAGGCTTATGTCAAATTGGCAACACCCGTCCCAATGAGGGGGGC	*P. inhibens* DSM 17395	Construction of the Δ*rmlA-D* mutant	Current study
1281	CTTCTATCGCCTTCTTGACGAGTTCTTCTGACCGTCCTGGGCTAACTGTGATGGCCGGGCTATAG	*P. inhibens* DSM 17395	Construction of the Δ*rmlA-D* mutant	Current study
1282	TACAAAAAAGCAGGCTCCGAATTCGCCCTTATGCTTAGCACCATGTCATCGATCACACTG	*P. inhibens* DSM 17395	Construction of the Δ*rmlA-D* mutant	Current study

Table S5: Primer list of bacterial genes for qRT-PCR.

Strain	Genetic locus	Gene name	Accession number	Forward Primer	Reverse Primer
D. shibae DFL-12	Chromosome	*recA*	DSHI_RS08365	CCGTAATCTCGA GAGCCTGC	GAAAGTTCCGG CAAGACCAC
	Chromosome	*gyrA*	DSHI_RS07515	GTGAAGCGGTA GAGCTGGTTC	CTCGACGAGAT CCCCTACCA
	191 kb and 126 kb plasmids	*virB8*	DSHI_RS18440 and DSHI_RS20035	TTCCGCTATGTG ACCGACC	CGATCCGGTTG ATGCTGAG
	191 kb and 126 kb plasmids	*virB4*	DSHI_RS18425 and DSHI_RS20020	CGATCTGACCG GCATTCTC	GTCACCAGCCA GTTCGACAG
	191 kb and 126 kb plasmids	*virD4*	DSHI_RS18490 and DSHI_RS20080	GAGCATGATCAT CACGGGC	CGGTGTCGGA CTTTGATTTC
P. inhibens P72	Chromosome	*recA*	PhaeoP72_RS07860	GCTGACACCCA AGTCGGAG	AGCCGAACATA ACGCCAATCT
	Chromosome	*gyrA*	PhaeoP72_RS07800	TCAGCTTATGTC GGGCTTCG	CGGTTCCTGAC CTCCTTCC
	18 kb and 57 kb plasmids	*virB8*	PhaeoP72_RS19740 and PhaeoP72_RS20905	GGGTTCGTCCA GACCTCATC	CCGGCTCGAT GTGGAAAT
	18 kb and 57 kb plasmids	*virB4*	PhaeoP72_RS19755 and PhaeoP72_RS20885	ACCGGCATTCT CGACAGC	GCGTATTGCGT CATCATCAC
	18 kb and 57 kb plasmids	*virD4*	PhaeoP72_03985 and PhaeoP72_RS20940	GCTTCGACCAAT AACGACACG	GAAGAGGCGG ATCAGCGG

Table S6: Plasmids used in the study.

Plasmid name	Origin	Comments
pBBR1MCS-5	Prof. Kenneth M. Peterson, Louisiana State University Medical Center, USA[82]	
pYDR1	Lab collection	Constructed using pBBR1MCS-5 and used in the current study as a vector carrying the sfGFP protein
pYDR3	Current study	Constructed using pYDR1 by replacing the sfGFP promoter with the 184 kb plasmid *virB* promoter for *P. inhibens* P72 reporter strain
pYDR10	Current study	Constructed using pYDR1 by replacing the sfGFP promoter with the 57 kb plasmid *virB* promoter for *P. inhibens* P72 reporter strain
pYDR6	Current study	Constructed using pYDR1 by replacing the sfGFP promoter with the 126 kb plasmid *virB* promoter for *D. shibae* DFL-12 reporter strain
pYDR7	Current study	Constructed using pYDR1 by replacing the sfGFP promoter with the 191 kb plasmid *virB* promoter for *D. shibae* DFL-12 reporter strain
pCR™8/GW/TOPO	Invitrogen	Cat#K250020
pYDR8	Lab collection	Constructed using pCR8/GW/TOPO vector (Invitrogen), and used in the current study as a vector carrying the kanamycin resistant cassette
pYDR9	Current study	Constructed using pYDR8 vector for creating the 65 kb intergenic mutant of *P. inhibens* DSM 17395
pYDR14	Current study	Constructed using pYDR8 vector for creating the *ΔexoY* mutant of *P. inhibens* DSM 17395
pYDR11	Current study	Constructed using pYDR8 vector for creating the *ΔrmlA-D* mutant of *P. inhibens* DSM 17395

Table S8. Quantification of donors, recipients, and transconjugants. Each conjugation donor-recipient pair from the conjugation assay described in figure 5 was plated on selective plates in serial dilutions, and quantified. The numbers indicate the colony-forming units per milliliter (CFU/ml). CFU values represent an average of two technical replicates for each biological sample for the donors and transconjugants (for conjugation efficiency calculations), and one technical replicate for the recipients. Dilution- indicates the dilution factor that was plated on the selective plates. ND- not detected and omitted from the average. Donor plasmid denoted as "p" is indicated by its size (Kb), recipients denoted as "chr", antibiotic resistance markers are indicated by gent for gentamicin-resistance and kana for kanamycin-resistance.

Donor strain	Recipient strain	Treatment	Biological replicate	Donor CFUs/ml	Dilution	Recipient CFUs/ml	Dilution	Transconjugants CFUs/ml
D. shibae DFL-12 $^{chr\text{-}gent}$	*P. inhibens* DSM 17395 $^{chr\text{-}kana}$	Control	1	6.83E+08	-5	2.50E+07	-4	4.55E+03
			2	7.70E+08	-5	3.20E+07	-4	4.30E+03
			3	7.65E+08	-5	2.00E+07	-4	7.53E+03
			4	6.10E+08	-5	2.40E+07	-4	3.05E+03
		Algal filtrates	1	5.70E+08	-5	4.20E+07	-4	8.40E+03
			2	6.40E+08	-5	2.70E+07	-4	5.90E+03
			3	5.65E+08	-5	4.40E+07	-4	7.75E+03
			4	6.60E+08	-5	3.30E+07	-4	8.80E+03
P. inhibens P72 $^{p57\text{-}kana}$	*D. shibae* DFL-12 $^{chr\text{-}gent}$	Control	1	6.00E+07	-4	2.90E+08	-5	6.35E+03
			2	5.75E+07	-4	4.10E+08	-5	4.75E+03
			3	5.90E+07	-4	2.80E+08	-5	4.80E+03
			4	6.70E+07	-4	2.60E+08	-5	4.55E+03
		Algal filtrates	1	1.00E+08	-4	5.40E+08	-5	9.65E+03
			2	1.50E+08	-4	6.70E+08	-5	8.30E+03
			3	9.13E+07	-4	4.80E+08	-5	9.00E+03
			4	9.33E+07	-4	5.25E+08	-5	2.25E+04
P. inhibens P72 $^{p57\text{-}kana}$	*M. algicola* DG898 $^{chr\text{-}gent}$	Control	1	6.65E+07	-4	4.90E+08	-5	2.05E+03
			2	7.50E+07	-4	ND	-5	1.20E+03
			3	7.85E+07	-4	4.50E+08	-5	1.45E+03
			4	7.35E+07	-4	4.80E+08	-5	3.05E+03
		Algal filtrates	1	9.05E+07	-4	3.00E+08	-5	1.06E+04
			2	7.95E+07	-4	3.60E+08	-5	1.14E+04
			3	8.60E+07	-4	2.80E+08	-5	1.35E+04
			4	8.05E+07	-4	4.60E+08	-5	2.08E+04
P. inhibens P72 $^{p57\text{-}kana}$	*P. inhibens* DSM 17395 $^{chr\text{-}gent}$	Control	1	1.20E+08	-4	1.20E+08	-5	1.38E+05
			2	9.00E+07	-5	7.00E+07	-5	1.02E+05
			3	9.00E+07	-4	1.00E+08	-5	1.40E+05
			4	9.00E+07	-5	1.30E+08	-5	2.50E+05
		Algal filtrates	1	1.00E+08	-4	1.60E+08	-5	3.45E+05
			2	1.60E+08	-5	1.20E+08	-5	5.65E+05
			3	1.60E+08	-5	1.40E+08	-5	4.40E+05
			4	2.10E+08	-5	1.50E+08	-5	6.00E+05
