# Supplementary material for: Algal exudates promote conjugation in marine Roseobacters
Source: mBio. 2024 Aug 27;15(10):e01062-24. doi: 10.1128/mbio.01062-24 (PMC11481893; doi:10.1128/mbio.01062-24)
Supplement: Table S7 — Roseobacteraceae genomes carrying complete T4SSs. [file mbio.01062-24-s0002.docx]

|  | | | |
| --- | --- | --- | --- |
|  | | | |
| **Table S7** | | | |
| Full list of Roseobacteraceae genomes carrying complete T4SSs available in the Intergrated Microbial Genomes @  Microbiomes (IMG/M) database | | | |
|  |  |  |  |
| Genome Name / Sample Name | IMG Genome ID (IMG Taxon ID) | T4SSs Genetic Loci |  |
| Phaeobacter gallaeciensis DSM 26640 | 2582580855 | Both plasmid and chromosome |  |
| Phaeobacter gallaeciensis P11 | 2791354902 | Both plasmid and chromosome |  |
| Phaeobacter gallaeciensis P63 | 2830026743 | Both plasmid and chromosome |  |
| Phaeobacter gallaeciensis P73 | 2830026743 | Both plasmid and chromosome |  |
| Phaeobacter gallaeciensis P75 | 2830026743 | Both plasmid and chromosome |  |
| Phaeobacter piscinae P14 | 2830026743 | Both plasmid and chromosome |  |
| Pseudosulfitobacter sp. DSM 107133 PIC-76 | 2987104877 | Both plasmid and chromosome |  |
| Roseovarius indicus DSM 26383 | 2987104877 | Both plasmid and chromosome |  |
| Ruegeria mediterranea CECT 7615 | 2832212542 | Both plasmid and chromosome |  |
| Sulfitobacter sp. N5S | 2832212542 | Both plasmid and chromosome |  |
| Falsihalocynthiibacter arcticus PAMC 20958 | 2728369471 | Chromosome |  |
| Leisingera aquimarina DSM 24565 | 2519899583 | Chromosome |  |
| Litoreibacter janthinus DSM 26921 | 2519899583 | Chromosome |  |
| Litoreibacter ponti DSM 100977 | 2616644820 | Chromosome |  |
| Phaeobacter gallaeciensis BS107 | 2616644820 | Chromosome |  |
| Phaeobacter gallaeciensis DSM 17395 | 2616644820 | Chromosome |  |
| Phaeobacter inhibens BS107 | 2616644820 | Chromosome |  |
| Phaeobacter inhibens P10/01/009 | 2806310875 | Chromosome |  |
| Phaeobacter inhibens P48/M21-2.3 | 2806310875 | Chromosome |  |
| Roseovarius halotolerans DSM 29507 | 2737471647 | Chromosome |  |
| Ruegeria sp. HKCCC2111 | 2737471647 | Chromosome |  |
| Ruegeria sp. HKCCC2112 | 2728369167 | Chromosome |  |
| Ruegeria sp. HKCCC2117 | 2802429340 | Chromosome |  |
| Shimia abyssi DSM 100673 | 2693429899 | Chromosome |  |
| Shimia biformata JCM 18818 | 2528768207 | Chromosome |  |
| Shimia sp. MIT1352 | 2528768207 | Chromosome |  |
| Sulfitobacter donghicola DSW-25, KCTC 12864 | 2617270870 | Chromosome |  |
| Sulfitobacter donghicola JCM 14565 | 2617270870 | Chromosome |  |
| Sulfitobacter mediterraneus SC1-11 | 2734482187 | Chromosome |  |
| Actibacterium atlanticum 22II-S11-Z10 | 2734482187 | Plasmid |  |
| Actibacterium lipolyticum CECT 8621 | 2513237353 | Plasmid |  |
| Actibacterium naphthalenivorans DSM 105040 | 2501004205 | Plasmid |  |
| Actibacterium ureilyticum LS-811 | 2501004205 | Plasmid |  |
| Aliiruegeria haliotis DSM 29328 | 2684622851 | Plasmid |  |
| Celeribacter baekdonensis B30 | 2832237884 | Plasmid |  |
| Celeribacter baekdonensis DSM 27375 | 2832237884 | Plasmid |  |
| Celeribacter baekdonensis LH4 | 2982263503 | Plasmid |  |
| Celeribacter baekdonensis strain LH4 reanalysis | 2982263503 | Plasmid |  |
| Celeribacter ethanolicus NH195 | 2982263503 | Plasmid |  |
| Celeribacter ethanolicus TSPH2 | 2622736501 | Plasmid |  |
| Celeribacter indicus DSM 27257 | 2775507242 | Plasmid |  |
| Celeribacter indicus P73 | 2693429899 | Plasmid |  |
| Celeribacter neptunius DSM 26471 | 2593339276 | Plasmid |  |
| Celeribacter persicus DSM 100434 | 2593339276 | Plasmid |  |
| Citreicella sp. 357 | 2622736446 | Plasmid |  |
| Dinoroseobacter shibae DFL-12, DSM 16493 | 2910745042 | Plasmid |  |
| Donghicola eburneus DSM 29127 | 2711768026 | Plasmid |  |
| Donghicola sp. KarMa | 2711768026 | Plasmid |  |
| Epibacterium sp. MM17-32 | 2622736430 | Plasmid |  |
| Epibacterium ulvae U95 | 2521172617 | Plasmid |  |
| Hwanghaeicola aestuarii DSM 22009 | 2512047087 | Plasmid |  |
| Jannaschia helgolandensis DSM 14858 | 2521172619 | Plasmid |  |
| Jannaschia marina SHC-163 | 2920479655 | Plasmid |  |
| Jannaschia rubra CECT 5088 | 2884316459 | Plasmid |  |
| Jannaschia rubra DSM 16279 | 2884316459 | Plasmid |  |
| Leisingera caerulea DSM 24564 | 2836636626 | Plasmid |  |
| Leisingera daeponensis DSM 23529 | 2836636626 | Plasmid |  |
| Leisingera sp. ANG59 | 2695420937 | Plasmid |  |
| Leisingera sp. JC1 | 2681812955 | Plasmid |  |
| Leisingera sp. NJS204 | 2617270836 | Plasmid |  |
| Litoreibacter ascidiaceicola DSM 100566 | 2681812954 | Plasmid |  |
| Litoreibacter halocynthiae DSM 29467 | 2734482799 | Plasmid |  |
| Litoreibacter meonggei DSM 29466 | 2890844935 | Plasmid |  |
| Litoreibacter roseus K6 | 2828431062 | Plasmid |  |
| Loktanella ponticola DSM 101064 | 2648501493 | Plasmid |  |
| Loktanella sp. 1ANDIMAR09 | 2791354982 | Plasmid |  |
| Loktanella sp. 22II-4b | 2648501806 | Plasmid |  |
| Loktanella sp. 3ANDIMAR09 | 2839538856 | Plasmid |  |
| Loktanella sp. Alg231-35 | 2751186040 | Plasmid |  |
| Loktanella sp. PT4BL | 2663762718 | Plasmid |  |
| Lutimaribacter pacificus CGMCC 1.10970 | 2663762718 | Plasmid |  |
| Lutimaribacter pacificus DSM 29620 | 2695420927 | Plasmid |  |
| Mameliella alba F15 | 2695420927 | Plasmid |  |
| Mameliella alba KD53 | 2788500142 | Plasmid |  |
| Mameliella alba L6M1-5 | 2788500142 | Plasmid |  |
| Mameliella atlantica DSM 104732 | 2791354926 | Plasmid |  |
| Mameliella phaeodactyli DSM 106184 | 2791354926 | Plasmid |  |
| Mameliella sp. LZ-28 | 2791354927 | Plasmid |  |
| Marinovum algicola CECT 5396 | 2830016464 | Plasmid |  |
| Marinovum algicola DG 898 | 2828474960 | Plasmid |  |
| Marinovum algicola DSM 10251 | 2891139733 | Plasmid |  |
| Maritimibacter alexandrii LZ-17 | 2891139733 | Plasmid |  |
| Maritimibacter alkaliphilus DSM 100037 | 2891139733 | Plasmid |  |
| Maritimibacter alkaliphilus HTCC2654 | 2833233958 | Plasmid |  |
| Maritimibacter harenae DP07 | 2833233958 | Plasmid |  |
| Maritimibacter sp. DP4N28-5 | 2513237001 | Plasmid |  |
| Maritimibacter sp. REDSEA-S40_B3 | 2513237001 | Plasmid |  |
| Marivita geojedonensis DSM 29432 | 2615840718 | Plasmid |  |
| Marivita hallyeonensis DSM 29431 | 2615840718 | Plasmid |  |
| Marivita sp. LZ-15-2 | 2890463227 | Plasmid |  |
| Muriiphilus fusiformis HY14 | 2890463227 | Plasmid |  |
| Nautella sp. ECSMB14104 | 2693429867 | Plasmid |  |
| Nautella sp. R17_0 | 2693429867 | Plasmid |  |
| Oceanicola sp. 22II-s10i | 2693429867 | Plasmid |  |
| Oceanicola sp. MCTG156(1a) | 648276686 | Plasmid |  |
| Pacificibacter marinus D2R04 | 648276686 | Plasmid |  |
| Palleronia abyssalis CECT 8504 | 2890434777 | Plasmid |  |
| Pelagibaca sp. ARS5 | 3001357244 | Plasmid |  |
| Pelagicola litoralis DSM 18290 | 3001357244 | Plasmid |  |
| Pelagimonas varians CECT 8663 | 2651870217 | Plasmid |  |
| Phaeobacter gallaeciensis P128 | 2651870217 | Plasmid |  |
| Phaeobacter gallaeciensis P129 | 2651870217 | Plasmid |  |
| Phaeobacter inhibens P24/M2-4.4 | 2728369485 | Plasmid |  |
| Phaeobacter inhibens P30/M4-3.1A | 2695421018 | Plasmid |  |
| Phaeobacter inhibens P54 | 2890224384 | Plasmid |  |
| Phaeobacter inhibens P66 | 2890224384 | Plasmid |  |
| Phaeobacter inhibens P70 | 2887557445 | Plasmid |  |
| Phaeobacter inhibens P72 | 2627854045 | Plasmid |  |
| Phaeobacter inhibens P74 | 2889183490 | Plasmid |  |
| Phaeobacter inhibens P78 | 2889183490 | Plasmid |  |
| Phaeobacter inhibens P80 | 2788500368 | Plasmid |  |
| Phaeobacter inhibens P83 | 2579779169 | Plasmid |  |
| Phaeobacter inhibens P88 | 3001050688 | Plasmid |  |
| Phaeobacter inhibens P92 | 2831527340 | Plasmid |  |
| Phaeobacter piscinae P13 | 2831527340 | Plasmid |  |
| Phaeobacter piscinae P18 | 2778261322 | Plasmid |  |
| Phaeobacter piscinae P23/M2-4.2 | 2778261322 | Plasmid |  |
| Phaeobacter piscinae P36 | 2574179735 | Plasmid |  |
| Phaeobacter piscinae P42 | 2832783666 | Plasmid |  |
| Phaeobacter piscinae P71 | 641380432 | Plasmid |  |
| Phaeobacter porticola P97 | **2510065029** | Plasmid |  |
| Phaeobacter sp. 11ANDIMAR09 | 2558309061 | Plasmid |  |
| Phaeobacter sp. O365 | 2558309061 | Plasmid |  |
| Phaeobacter sp. S60 | 2814123108 | Plasmid |  |
| Pseudoruegeria marinistellae SF-16 | 2814123108 | Plasmid |  |
| Pseudosulfitobacter pseudonitzschiae H3 | 2814123083 | Plasmid |  |
| Roseicyclus mahoneyensis DSM 16097 | 2814123089 | Plasmid |  |
| Roseivivax halodurans JCM 10272 | 2814123091 | Plasmid |  |
| Roseivivax halotolerans DSM 15490 | 2814123091 | Plasmid |  |
| Roseivivax isoporae LMG 25204 | 2814123091 | Plasmid |  |
| Roseivivax sediminis DSM 26472 | 2814123117 | Plasmid |  |
| Roseivivax sp. THAF197b | 2814123117 | Plasmid |  |
| Roseivivax sp. THAF30 | 2814123081 | Plasmid |  |
| Roseivivax sp. THAF40 | 2814123081 | Plasmid |  |
| Roseobacter denitrificans DSM 7001 | 2814123081 | Plasmid |  |
| Roseobacter denitrificans FDAARGOS_309 | 2841416095 | Plasmid |  |
| Roseobacter denitrificans OCh 114 | 2814123246 | Plasmid |  |
| Roseobacter sp. MED193 | 2814123342 | Plasmid |  |
| Roseobacter sp. N2S | 2814123342 | Plasmid |  |
| Roseobacter sp. SAT8 | 2814123342 | Plasmid |  |
| Roseovarius amoyensis GCL-8 | 2814123329 | Plasmid |  |
| Roseovarius atlanticus R12b | 2814123329 | Plasmid |  |
| Roseovarius azorensis DSM 100674 | 2814123348 | Plasmid |  |
| Roseovarius confluentis SAG6 | 2814123349 | Plasmid |  |
| Roseovarius indicus B108 cultivar:MA | 2814123349 | Plasmid |  |
| Roseovarius litoreus DSM 28249 | 2814123350 | Plasmid |  |
| Roseovarius lutimaris DSM 28463 | 2814123350 | Plasmid |  |
| Roseovarius mucosus DSM 17069 | 2814123350 | Plasmid |  |
| Roseovarius mucosus SMR3 | 2814123350 | Plasmid |  |
| Roseovarius nanhaiticus CGMCC 1.10961 | 2814123315 | Plasmid |  |
| Roseovarius nanhaiticus DSM 29590 | 2814123315 | Plasmid |  |
| Roseovarius nitratireducens TFZ | 2814123315 | Plasmid |  |
| Roseovarius sp. 217 | **2814123347** | Plasmid |  |
| Roseovarius sp. 22II1-1F6A | **2814123347** | Plasmid |  |
| Roseovarius sp. A-2 | 2814123299 | Plasmid |  |
| Roseovarius sp. AK1035 | 2814123299 | Plasmid |  |
| Roseovarius sp. EC-HK134 RV134 | 2814123299 | Plasmid |  |
| Roseovarius sp. EC-SD190 RV420 | 2814123300 | Plasmid |  |
| Roseovarius sp. HI0049 | 2814123300 | Plasmid |  |
| Roseovarius sp. M141 | 2814123300 | Plasmid |  |
| Roseovarius sp. MBR-78 | 2814123328 | Plasmid |  |
| Roseovarius sp. MCTG156(2b) | 2814123328 | Plasmid |  |
| Roseovarius sp. THAF27 | 2814123328 | Plasmid |  |
| Roseovarius sp. THAF8 | 2814123328 | Plasmid |  |
| Roseovarius sp. THAF9 | 2814123277 | Plasmid |  |
| Roseovarius sp. TM1035 | 2814123277 | Plasmid |  |
| Roseovarius spongiae HN-E21 | 2814123277 | Plasmid |  |
| Roseovarius tolerans DSM 11457 | 2814123341 | Plasmid |  |
| Rubellimicrobium aerolatum DSM 19297 | 2814123341 | Plasmid |  |
| Rubellimicrobium rubrum YIM 131921 | 2814123341 | Plasmid |  |
| Ruegeria marina CGMCC 1.9108 | 2814123287 | Plasmid |  |
| Ruegeria sp. EL01 | 2814123287 | Plasmid |  |
| Ruegeria sp. HKCCA5463 | 2814123287 | Plasmid |  |
| Ruegeria sp. HKCCD6119 | 2706794654 | Plasmid |  |
| Ruegeria sp. R13_0 | 2706794654 | Plasmid |  |
| Ruegeria sp. R14_0 | 2775506953 | Plasmid |  |
| Ruegeria sp. THAF33 | 2775506953 | Plasmid |  |
| Ruegeria sp. THAF57 | 2775506953 | Plasmid |  |
| Ruegeria sp. TM1040 | 2775506957 | Plasmid |  |
| Sagittula marina DSM 102235 | 2814123288 | Plasmid |  |
| Sagittula sp. P11 | 2775506956 | Plasmid |  |
| Sagittula stellata E-37 | 2775506956 | Plasmid |  |
| Salipiger abyssi JLT2014 | 2775506955 | Plasmid |  |
| Salipiger aestuarii DSM 22011 | 2775506955 | Plasmid |  |
| Salipiger pacificus CGMCC 1.3455 | 2775506955 | Plasmid |  |
| Salipiger pacificus DSM 26894 | 2814123285 | Plasmid |  |
| Salipiger profundus DSM 27508 | 2814123285 | Plasmid |  |
| Salipiger profundus JLT2016 | 2814123285 | Plasmid |  |
| Salipiger sp. PrR002 | 2814123257 | Plasmid |  |
| Salipiger sp. PrR003 | 2718218026 | Plasmid |  |
| Salipiger sp. PrR004 | 2645728037 | Plasmid |  |
| Salipiger sp. PrR007 | 2645728037 | Plasmid |  |
| Salipiger thiooxidans DSM 10146 | 2687453711 | Plasmid |  |
| Shimia marina CECT 7688 | 2687453711 | Plasmid |  |
| Shimia marina DSM 26895 | 2675903626 | Plasmid |  |
| Shimia sp. WX04 | 2675903626 | Plasmid |  |
| Sulfitobacter alexandrii AM1-D1 | 2675903626 | Plasmid |  |
| Sulfitobacter algicola 1151 | 2675903626 | Plasmid |  |
| Sulfitobacter brevis DSM 11443 | 2718217653 | Plasmid |  |
| Sulfitobacter delicatus DSM 16477 | 2994677068 | Plasmid |  |
| Sulfitobacter geojensis DSM 101063 | 2994677068 | Plasmid |  |
| Sulfitobacter geojensis MM-124 | 2728368996 | Plasmid |  |
| Sulfitobacter indolifex HEL-45 | 3001084934 | Plasmid |  |
| Sulfitobacter litoralis DSM 17584 | 3001084934 | Plasmid |  |
| Sulfitobacter noctilucae NB-68 | 2585427828 | Plasmid |  |
| Sulfitobacter noctilucicola DSM 101015 | 2844187691 | Plasmid |  |
| Sulfitobacter noctilucicola NB-77 | 2844187691 | Plasmid |  |
| Sulfitobacter pontiacus DSM 10014 | 2756170271 | Plasmid |  |
| Sulfitobacter pseudonitzschiae DSM 26824 | 2563367175 | Plasmid |  |
| Sulfitobacter pseudonitzschiae SMR1 | 2675903164 | Plasmid |  |
| Sulfitobacter sp. 20_GPM-1509m | 2609459988 | Plasmid |  |
| Sulfitobacter sp. CB2047 | 2634166317 | Plasmid |  |
| Sulfitobacter sp. D7 | 2884205492 | Plasmid |  |
| Sulfitobacter sp. DFL-14 | 2884205492 | Plasmid |  |
| Sulfitobacter sp. DFL-23 | 2884222058 | Plasmid |  |
| Sulfitobacter sp. EhC04 | 2884222058 | Plasmid |  |
| Sulfitobacter sp. EL44 | 2884226019 | Plasmid |  |
| Sulfitobacter sp. HGT1 | 2884226019 | Plasmid |  |
| Sulfitobacter sp. HI0023 | 2693429868 | Plasmid |  |
| Sulfitobacter sp. HI0040 | 2841198529 | Plasmid |  |
| Sulfitobacter sp. R18_2 | 639633056 | Plasmid |  |
| Sulfitobacter sp. THAF37 | 638341182 | Plasmid |  |
| Thalassobacter stenotrophicus CECT 5294 | 638341182 | Plasmid |  |
| Thalassobacter stenotrophicus DSM 16310 | 2838011328 | Plasmid |  |
| Thalassobius autumnalis 5120 | 2838011328 | Plasmid |  |
| Thalassobius autumnalis CECT 5118 | 2838011328 | Plasmid |  |
| Thalassobius mediterraneus CECT 5383 | 2788500504 | Plasmid |  |
| Thalassobius mediterraneus DSM 16398 | 2788500504 | Plasmid |  |
| Thalassobius taeanensis DSM 22007 | 2843396798 | Plasmid |  |
| Thalassococcus halodurans DSM 26915 | 2791355032 | Plasmid |  |
| Tranquillimonas rosea DSM 23042 | 2791355032 | Plasmid |  |
| Tropicibacter naphthalenivorans CECT 7648 | 2693429884 | Plasmid |  |
| Tropicibacter naphthalenivorans DSM 19561 | 2883117184 | Plasmid |  |
| Tropicibacter phthalicicus CECT 8649 | 2728369466 | Plasmid |  |
| Tropicibacter phthalicicus DSM 26923 | 2731957848 | Plasmid |  |
| Tropicibacter sp. LMIT003 | 2619618997 | Plasmid |  |
| Tropicibacter sp. R15_0 | 8002761742 | Plasmid |  |
| Actibacterium sp. EMB200-NS6 | 8002761742 | Unknown |  |
| Phaeobacter italicus CECT 7321 | 2700988728 | Unknown |  |
| Pseudooceanicola flagellatus CGMCC 1.12644 | 2622736530 | Unknown |  |
| Pseudoruegeria sp. GL-11-2 | 2622736530 | Unknown |  |
| Salipiger abyssi SY10-16 | 2518285587 | Unknown |  |
| Salipiger mangrovisoli 6D45A | 2772190778 | Unknown |  |
| Salipiger pallidus CGMCC 1.15762 | 2667528179 | Unknown |  |
| Sulfitobacter mediterraneus SC1-11 | 2681812935 | Unknown |  |
| Sulfitobacter sp. BSw21498 | 2840908651 | Unknown |  |
|  |  |  |  |
